# Supplementary material for: Chirality-Induced Orbital-Angular-Momentum Selectivity in Electron Transmission and Scattering
Source: J Chem Theory Comput. 2025 Dec 31;22(1):20–9. doi: 10.1021/acs.jctc.5c01410 (PMC12805573; doi:10.1021/acs.jctc.5c01410)
Supplement: Supplementary file 1 [file ct5c01410_si_001.pdf]

Supplemental Information for
Chirality-Induced Orbital-Angular-Momentum Selectivity in Electron Transmission and Scattering

Yun Chen,[1] Oded Hod[1,*], Joel Gersten,[2] and Abraham Nitzan[1,3]

[1] Department of Physical Chemistry, School of Chemistry, The Raymond and Beverly Sackler Faculty of Exact Sciences and The Sackler Center for Computational Molecular and Materials Science, Tel Aviv University, Tel Aviv 6997801, Israel

[2] Department of Physics, City College of the City University of New York, New York, New York 10031, USA

[3] Department of Chemistry, University of Pennsylvania, 231 South 34th Street, Philadelphia, Pennsylvania 19104, USA

[*]Corresponding author email: odedhod@tauex.tau.ac.il

This supplementary information document includes the following sections:

Section 1. Helix size-dependence of orbital angular momentum polarization 2

Section 2. Effect of dissipative scatterer motion 5

Section 3. Spatial resolution of scattering wavepackets during bond collision 8

Section 4. Effect of scattering surface model thickness 10

Section 5. Orbital angular momentum-based spatial resolution via surface scattering 12

Section 6. Dependence of wavepacket deflection on the repulsive potential parameters..... 14

Supplementary Movie Legends:

SI movie 1: Electronic orbital angular momentum polarization by a charged helical potential

SI movie 2: Collision-induced electron orbital angular momentum reversal

SI movie 3: Asymmetric electron diffraction from a single scatterer

SI movie 4: Surface scattering of a spinning electronic wavepacket

SI movie 5: Phase-resolved surface scattering of a spinning electronic wavepacket

Section 1. Helix size-dependence of orbital angular momentum polarization

An important parameter that influences the polarization of orbital angular momentum (OAM) of electrons traversing a helical potential is the ratio between the initial wavepacket width and the radius of the charged helical chain, $\tau = \sigma/R_h$. To evaluate the effect of this parameter on OAM polarization, we repeated some of the simulations for Gaussian electronic wavepackets of initial width of $\sigma = 1$ Å traversing left-handed charged helical chains of radii $R_h = 1,\ 2,\ 5,$ and 10 Å. This R_h range matches characteristic radii of chiral molecules commonly used in CISS experiments, including: helicenes (~3 Å)[1-3], α-helical peptides (~6 Å)[4-11], and DNA (~10 Å)[7, 8, 12, 13] (see Table S1).

Table S1. Comparison of structural parameters of DNA, α helical peptides, and helicenes employed in CISS experiments with our chiral helical potential model parameters.

System	Helix radius* (Å)	Pitch length (Å)	Molecular length (Å)
DNA[14, 15]	~10	~34	68 – 170
α helical peptides[16, 17]	~6	~5.4	16 – 54
Helicenes[18]	~3	~3.2	3.56 – 5.16
Our models	1 – 10	5 – 10	10 – 20

* **The radii for realistic molecules correspond to their backbone radii.**

Fig. S1 presents snapshots of the wavepacket evolution at $t = 0$ and 0.4 fs, generally demonstrating that as τ decreases the angular momentum accumulated by the wavepacket reduces.

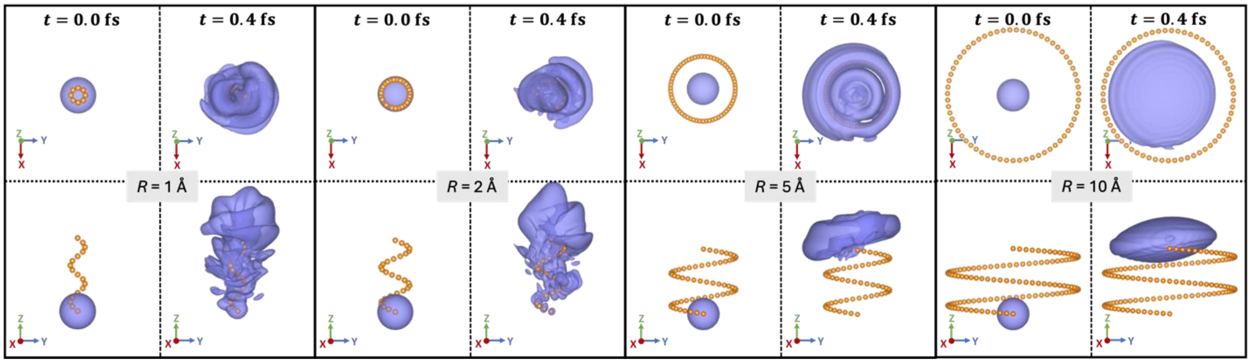

Figure S1. Snapshots, taken at $t = 0$ and $0.4\ fs$, from the quantum dynamics of Gaussian electronic wavepackets of initial width of $\sigma = 1$ Å, driven through left-handed point charge helical chains via an external vertical electric field of $E_z = -5\ V/\text{Å}$. The point charges carry a charge of 1.0 $a.u.$ each and are separated by an arclength of 1 Å. Four different left-handed helices are considered with radii of $R_h = 1$ (two leftmost columns), 2 (two left-middle columns), 5 (two right-middle columns), and 10 Å (two rightmost columns) and two pitches, each of $P_h = 5$ Å. Top and side views are presented in the top and bottom panels, respectively.

Fig. S2 presents the vertical angular momentum (with respect to the central helix axis) acquired by the wavepacket while traversing the various charged helical chains. The results support the general claim made above, where the electron propagating through the $R_h = 10$ Å helix barely carries any OAM upon exit, the one crossing the $R_h = 5$ Å helix carries somewhat higher OAM, and those traveling along the $R_h = 2$ and 1 Å helices acquire sizable OAM during their motion through the charged helix. Notably, for $R_h = 2$ Å higher OAM is gained than for $R_h = 1$ Å. This turnover is to be expected as in the limit $\tau \gg 1$ the geometry of the charged helical chain influences mainly the inner core of the wavepacket. The observed reduction in electronic OAM upon departure from the helix results from the back attraction of the wavepacket towards the charged chain. In the absence of the driving field, this would cause the wavepacket to reverse its rotational sense while re-entering the helix. In the presence of the field, it is manifested as OAM reduction.

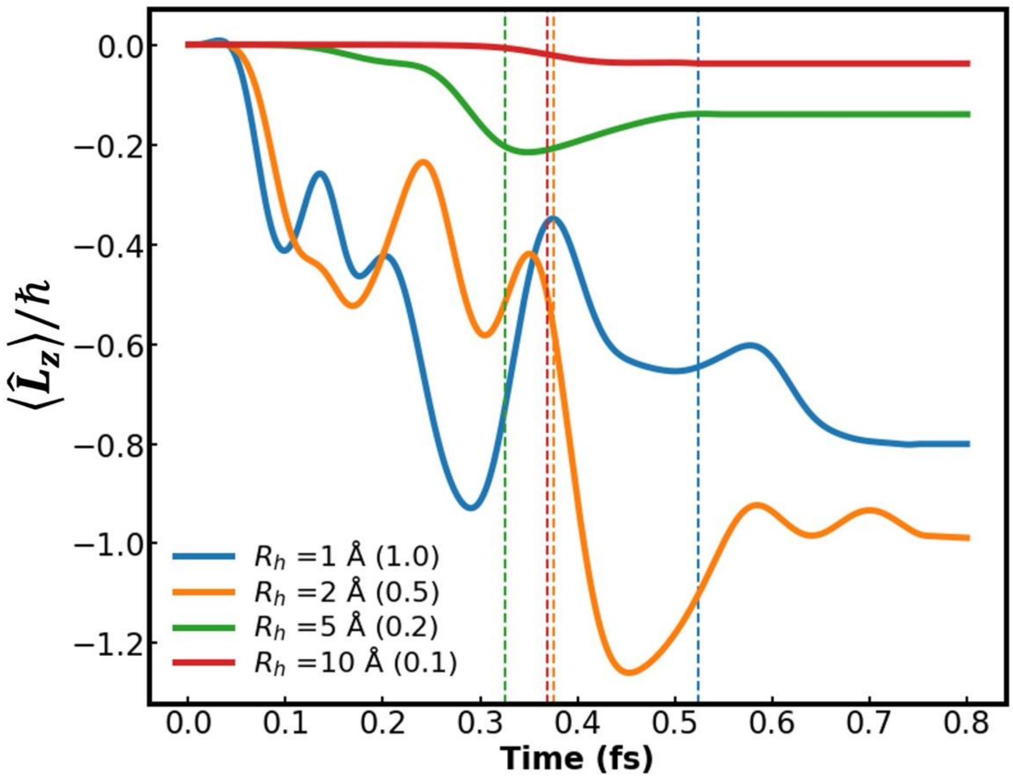

Figure S2. Time evolution of the vertical angular momentum expectation value, $\langle \hat{L}_z \rangle / \hbar\,(t)$, of a Gaussian wavepacket of initial width $\sigma_x = \sigma_y = \sigma_z = 1$ Å traversing left-handed helices of $R_h = 1$ (blue line), 2 (orange line), 5 (green line), and 10 (red line) Å. All other simulation parameters are the same as in Fig. S1. The ratios between the initial wavepacket width and the helix radii, $\tau = \sigma / R_h$, are given in parentheses. The vertical dashed lines indicate the time at which the vertical position expectation value of the wavepacket, $\langle \hat{z} \rangle(t)$, crosses the top of the helix.

Section 2. Effect of dissipative scatterer motion

The point scatterers used in the main text to mimic the reflecting wall may be viewed as surface atoms. While we considered them to be fixed in space (i.e., carrying an infinite mass), in practice they may recoil due to the colliding electron thus dissipating the wavepacket energy and influencing its dynamics. To evaluate to what extent this may influence the spatial separation of wavepackets carrying opposite OAM, we simulated the diffraction of a two-dimensional Gaussian electronic wavepacket from a single classical scatterer of mass 1.66054×10^{-27} kg (the proton mass), anchored at a vertical distance of 47.75 Å from the minimum of a Lennard-Jones (LJ) potential (see Eq. [3] of the main text) with a 2D spring of constant $k_a = 6.58 \times 10^{-3}$ nN/Å (see inset of Fig. S3a), corresponding to a frequency of 1 THz, typical to molecular vibrations. Ehrenfest dynamics[19] was used to perform the mixed quantum-classical scattering simulations, where the electronic wavepacket follows the time-dependent Schrödinger equation (TDSE):

$$i\hbar \frac{\partial \Psi(r,t)}{\partial t} = \left[-\frac{\hbar^2}{2m_e}\nabla^2 + V_{LJ}(y) + \sum_i V_{\mathrm{P}}\big(\boldsymbol{r};\boldsymbol{r}_i(t)\big) \right] \Psi(\boldsymbol{r},t), \quad [1]$$

where $V_{\mathrm{P}}\big(\boldsymbol{r};\boldsymbol{r}_i(t)\big)$ is the repulsive potential experienced by the electronic wavepacket due to a scatterer of instantaneous location $\boldsymbol{r}_i(t)$ (see Eq. [6] of the main text), and the classical scatterers follow Newton's equations of motion:

$$\ddot{\boldsymbol{r}}_i = -\frac{1}{M_{\mathrm{s}}}\left(\boldsymbol{\nabla}_i V_{\mathrm{s-wall}} + \langle \Psi(\boldsymbol{r},t) | \boldsymbol{\nabla}_i V_{\mathrm{P}}(\boldsymbol{r};\boldsymbol{r}_i) | \Psi(\boldsymbol{r},t) \rangle\right) - \eta v_i(t), \quad [2]$$

where $V_{\mathrm{s-wall}}$ is the scatter-wall harmonic spring potential, η is a viscous damping coefficient and $v_{\mathrm{i}} = \dot{x}_i$ is the classical velocity of scatterer i. Since we are concerned with the energy dissipation through the energy transfer from the electronic wave packet to the scatterer, we set $\eta = 0$, ignoring the dissipative behavior of the scatterer itself in the classical dynamics, with negligible effect on the results. The fourth order-Runge-Kutta propagation scheme was used to integrate the TDSE and the Verlet algorithm was used to propagate Newton's equations of motion, both with a fixed time step of $dt = 10^{-5}$ fs.

Fig. S3a compares the expectation values of the lateral ($\langle x \rangle$) and vertical ($\langle y \rangle$) coordinates of an electronic wavepacket of initial width $\sigma = 1$ Å, carrying angular momentum of $m = +1$, and given an initial vertical center-of-mass velocity of v_y =30 Å/fs, during collision with a fixed scatterer (blue solid line), a free-standing scatterer (orange dashed line), and a scatterer anchored

to the minimum of the vertical LJ potential via a spring (green dotted line). The repulsion strength of the scattering potential was taken to be $A = 200$ eV and the interaction length was chosen as $b = 1$ Å. As can be seen, no visible difference between the three electronic wavepacket trajectories is observed, and negligible displacement of the scatterer is recorded in the free and spring-anchored cases (Fig. S3b, c). This clearly demonstrates that dissipation due to scatterer recoil has no significant effect on the OAM spatial separation in the present case, mainly because of the large mass difference between the electron and the scattering particle.

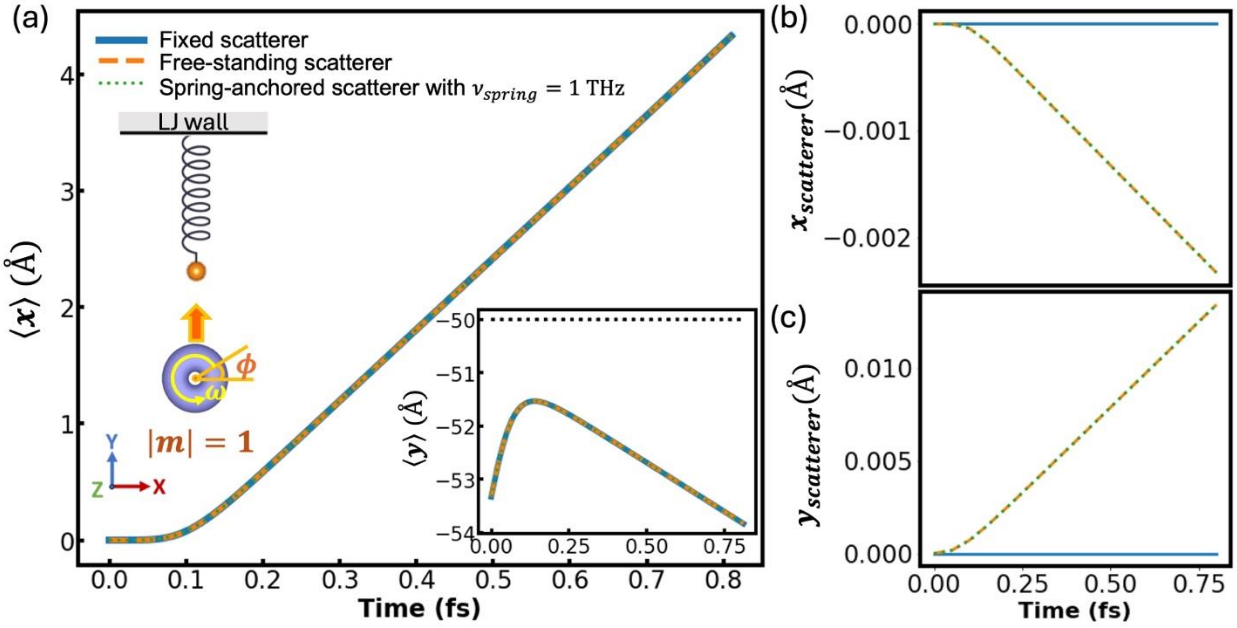

Figure S3. (a) Temporal evolution of the lateral ($\langle x \rangle$, main panel) and vertical ($\langle y \rangle$, inset) components of the position expectation value of an electronic wavepacket colliding with a single fixed (full blue line), free standing (dashed orange line), or spring anchored (dotted green line) scatterer. (b), (c) Lateral (x) and vertical (y) displacement of the classical scatterer during collisions, respectively.

While energy dissipation via phononic degrees of freedom has a negligible effect on spinning electronic wavepacket scattering, electronic friction may significantly contribute to OAM-based spatial resolution. To demonstrate this, we repeated our simulations for a flexible two-row electronic scatterer wall, augmented by a rear LJ potential, where each scatterer is assigned the electron mass, and is anchored to its equilibrium location by a two-dimensional spring of the same vibrational frequency of 1 THz, so as to change only one simulation parameter. We note that the wavepacket scattering trajectory is relatively insensitive to the choice of spring vibration frequency

in this case. Panels (a) and (b) in Figure S4 display simulation snapshots (electron density probability and scatterer locations at $t = 0.05, 0.3$ and 0.6 fs) for head-on collisions between an electronic Gaussian wavepacket, carrying an initial angular momentum of $m = +1$ and having an initial width of $\sigma_x = \sigma_y =3$ Å, and scatterers of electron or proton masses, respectively. Panels (c) and (d) in Fig. S4 present the corresponding time evolution of the total electronic wavepacket energy and the expectation values of the lateral, $\langle x \rangle$ (main panel), and vertical, $\langle y \rangle$ (inset), wavepacket coordinates, respectively. Blue and green lines represent results obtained for light and heavy scatterers, respectively. For the light scatterers, we observe substantial deformation of the scattering surface (see Fig. S4a) and significant energy transfer from the electronic wavepacket to the scatterers (see Fig. S4c), leading to enhanced lateral deflection of the electronic wavepacket (Fig. S4d). These findings suggest that electronic friction may play a central role in vortex electron scattering and spatial resolution, an interesting direction that will be further pursued in our future studies.

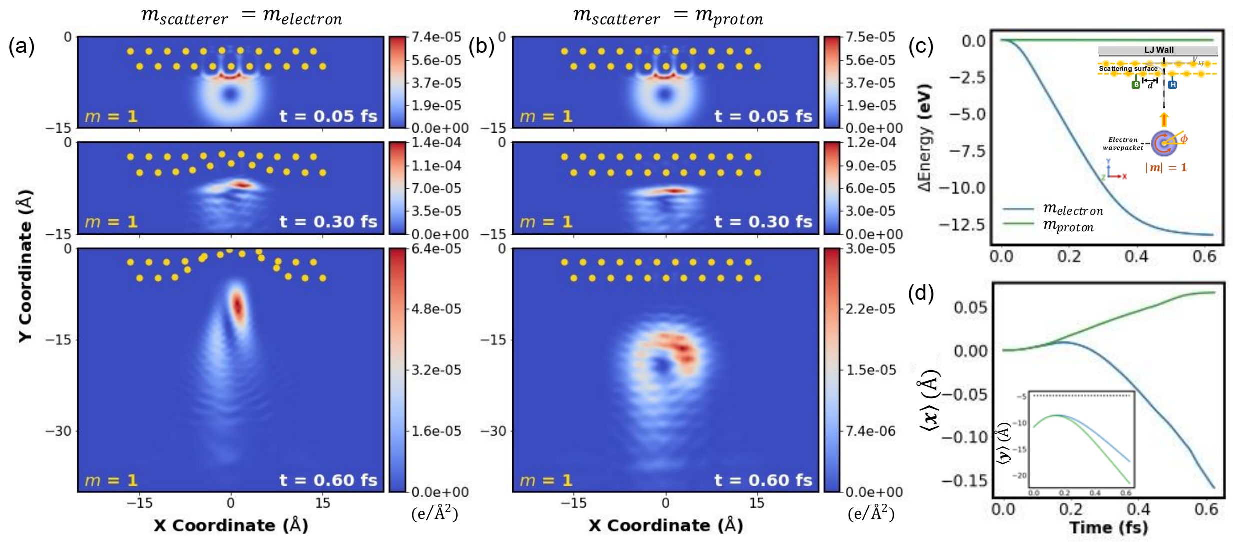

Figure S4. (a) [(b)] Snapshots taken from head collision simulations of a Gaussian electronic wavepacket of initial width $\sigma = 3$ Å, positioned -10.9 Å below two (laterally shifted) scatterer rows of masses $m_{scatterer} = m_{electron}$ (left column) and $m_{scatterer} = m_{proton}$ (right column) and given OAM of $m = +1$ and a vertical velocity of $v_y = 30$ Å/fs towards the surface. The snapshots are taken at $t = 0.05$ (top row), 0.3 (middle row), and 0.6 fs (bottom row). All other simulation parameters are the same as those used to obtain Fig. S3. (c) Total energy variation of the electronic wavepackets during the collision process. The inset provides a schematic illustration of the collision model. (d) Time evolution of the expectation values of the lateral, $\langle x \rangle$, and vertical $\langle y \rangle$ electronic wavepacket coordinates.

Section 3. Spatial resolution of scattering wavepackets during bond collision

In Figs. 6 and 7 of the main text we presented simulation results of head-on collision of a Gaussian electronic wavapacket with a scattering surface, where the wavepacket is initially positioned with its center-of-mass directly in front of one of the scattering particles and given initial vertical velocity. To evaluate the effect of the initial lateral position of the wavepacket on the scattering process and the resulting spatial orbital angular momentum resolution, we repeated some of the simulations for bond collisions, where the center-of-charge of the wavepacket is initially positioned in between two scattering sites.

Figure S5 presents simulation snapshots (at $t = 0, 0.05$, and 0.7 fs) of bond collision scattering of electronic Gaussian wavepackets carrying an initial angular momentum of $m = +1$ and having initial widths of $\sigma_x = \sigma_y = 1$, 3, and 5 Å. For all three cases the reflected wavepackets resemble those of the head-on collision case (Fig. 6b of the main text). Furthermore, when the initial wavepacket width is comparable to, or larger than, the inter-scatterer spacing (3 Å), the scattering spatial asymmetry is insensitive to its initial lateral position (see Fig. 6c of the main text). However, when the initial wavepacket width is smaller than the surface periodicity local interactions cause the reflected wavepacket to laterally deflect in the opposite direction, compared to head-on collision.

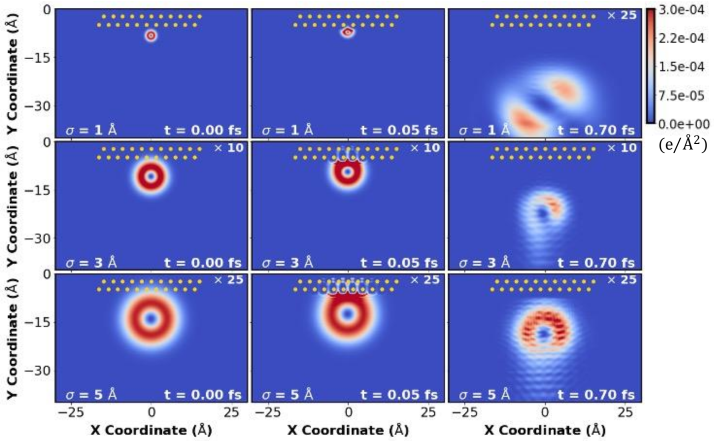

Figure S5. Snapshots taken from bond collision simulations of Gaussian electronic wavepackets of initial widths $\sigma =$ 1 (top row), 3 (middle row), or 5 Å (bottom row), positioned -8.2, -10.9, or -14.0 Å below the scatterers line, respectively, and given OAM of $m = +1$ and a vertical velocity of $v_y = 30$ Å/fs towards the surface. The snapshots are taken at $t = 0$ (left column), 0.05 (middle column), and 0.7 fs (right column). All other simulation parameters are the same as in Fig. 6 of the main text.

When an impurity scatterer is introduced at a surface bond position, the scattered electronic wavepackets and the corresponding orbital angular momentum spatial resolution (see Fig. S6) are found to be nearly indistinguishable from those obtained when the scatterer is positioned above a surface scatterer (see Fig. 7a of the main text), for all three initial Gaussian wavepacket widths considered. This indicates the robustness of our results.

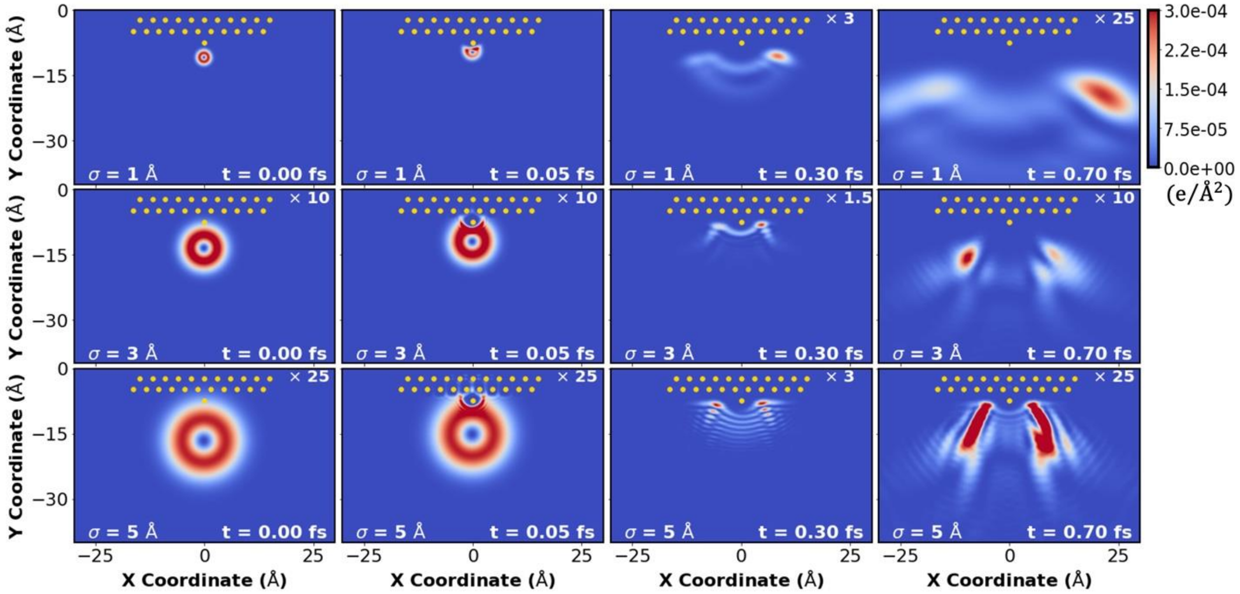

Figure S6. Snapshots taken from head-on collision simulations of 2D Gaussian electronic wavepackets of initial widths $\sigma = 1$ (top row), 3 (middle row), or 5 Å (bottom row), positioned -10.78, -13.45, or -16.6 Å below the scatterers line, respectively, and given OAM of $m = +1$ and a vertical velocity of $v_y = 30$ Å/fs towards an impurity scatterer positioned at a surface bond location. The snapshots are taken at $t = 0$ (left column), 0.05 (second column), 0.3 (third column), and 0.7 fs (right column). All other simulation parameters are the same as in Fig. 7 of the main text.

Section 4. Effect of scattering surface model thickness

In Fig. 6 of the main text, we employed two scatterer rows to mimic the atomic structure of a scattering surface, augmented by a rear smooth LJ vertical wall that accounted for bulk reflection. To validate that a two-scatterer row model is sufficient, we repeated some of the calculations with single and four scatterer row models. Figure S7 compares the temporal evolution of the lateral ($\langle x \rangle$, main panel) and vertical ($\langle y \rangle$, inset) components of the position expectation value of an electronic wavepacket of initial width $\sigma = 1$ Å, carrying angular momentum of $m = +1$, and given an initial vertical center-of-mass velocity of v_y =30 Å/fs, during collision with a single scatterer row (blue lines), two scatterer rows (orange lines), and four scatterer rows (green lines), augmented by a vertical LJ wall with $\varepsilon_{LJ} = 0.05$ eV (see Eq. [3] of the main text), the minimum of which is positioned at the rear scatterer row. The inter-scatterer distance and inter-row distance are both taken to be 3 Å and every two adjacent scatterer rows are laterally shifted by half the lattice constant with respect to each other. The repulsion strength of the scattering potential was taken to be $A = 200$ eV and the interaction length was chosen as $b = 1$ Å. All three scattering surface models provide the same qualitative behavior and the two- and four-row models give essentially the same quantitative center-of-charge trajectories. These results support the adequacy of the two-scatterer row model adopted in the main text.

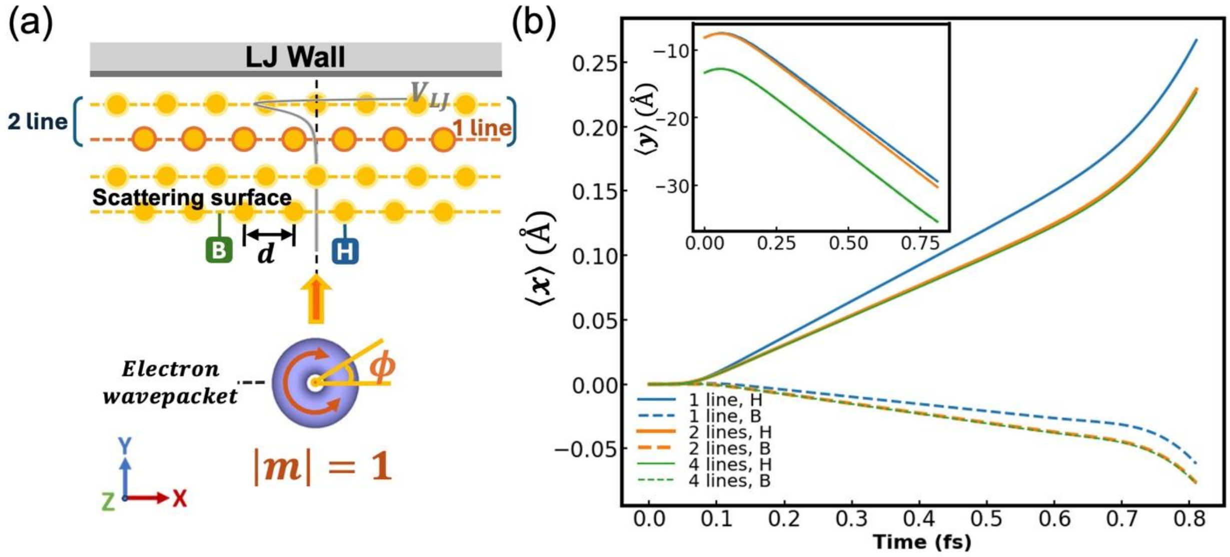

Figure S7. (a) Schematic illustration of the scattering of a 2D Gaussian electronic wavepacket from one-, two- and four-row fixed scatterer surfaces. The scatterer rows are laterally shifted with respect to each other by half a lattice constant, $0.5d = 1.5$ Å. A vertical LJ potential with $\varepsilon_{LJ} = 0.05\ eV$ is introduced to mimic scattering from bulk layers, with its minimum located at the inner scatterer row ($y_{min} = -2.25$ Å, grey curve). The center-of-charge of the wavepacket is initially located either in front of one of the first-row scatterers (head-on collision, marked as H) or in between two such scatterers (bond collision, marked as B). (b) Temporal evolution of the lateral ($\langle x \rangle$, main panel) and vertical ($\langle y \rangle$, inset) components of the position expectation value of an electronic wavepacket colliding with a single scatterer row (blue lines), two scatterer rows (orange lines), or four scatterer rows (green lines) augmented by a rear vertical LJ wall. Full and dashed lines correspond to initial conditions where the center of mass of the wave packet is located directly in front of a scattering site (head-on collision, marked as H) and in-between scattering sites (bond collision, marked as B).

Section 5. Orbital angular momentum-based spatial resolution via surface scattering

In the main text we presented results for surface scattering of 2D Gaussian electronic wavepackets carrying initial angular momentum of $m = +1$. To demonstrate the scattering-induced orbital angular momentum-based spatial resolution of wavepackets carrying opposite initial angular momenta, we compare in Fig. S8 the time evolution of the lateral position expectation value component, $\langle x \rangle$, of wavepackets of widths $\sigma_x = \sigma_y = 1$ Å with initial angular momenta of $m = +1$, 0, and -1, and given a center of charge velocity of v_y =30 Å/fs towards a rigid scatterer surface in a head-on collision trajectory. The $m = 0$ wavepacket reflects symmetrically from the scattering surface with no lateral deflection, whereas the $m = \pm 1$ wavepackets deflect to the right or to the left depending on their initial rotational sense. The mirror symmetric scattering trajectories of the latter lead to orbital angular momentum-based spatial resolution. A qualitatively similar picture, but with much more pronounced spatial resolution, arises for the impurity scattering scenario (see Fig. S9).

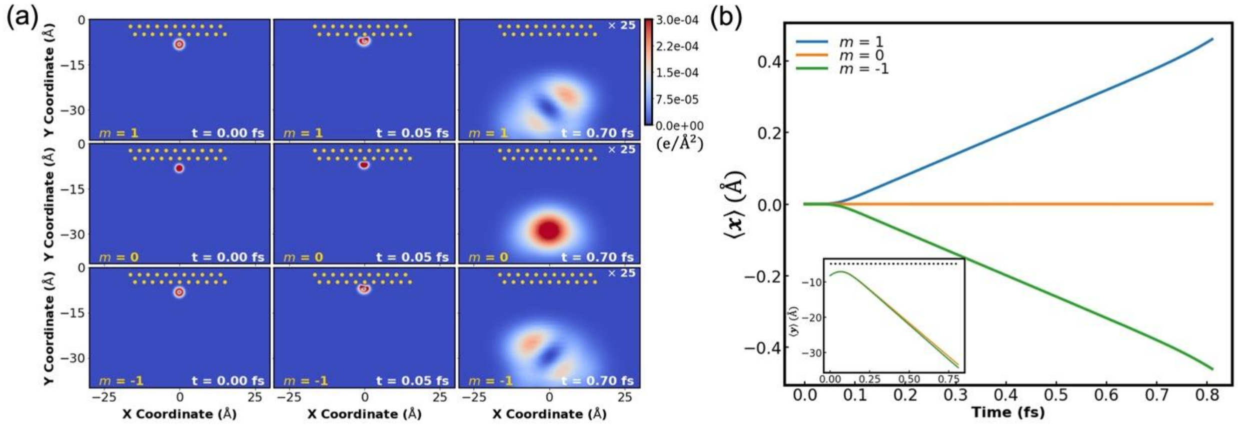

Figure S8. (a) Comparison of head-on collision scattering trajectories of 2D Gaussian electronic wavepackets carrying initial angular momenta of $m = +1$ (top row), 0 (middle row), and -1 (bottom row). Snapshots are taken at simulation times of $t = 0$ (left column), 0.05 (middle column), and 0.7 (right column) fs. All other simulation parameters are the same as those used for the $\sigma = 1$ Å wavepacket in main text Fig. 6. (b) Temporal evolution of the lateral ($\langle x \rangle$, main panel) and vertical ($\langle y \rangle$, inset) components of the position expectation value of the electronic wavepackets depicted in panel (a) with $m = +1$ (blue lines), 0 (orange lines) and -1 (green lines).

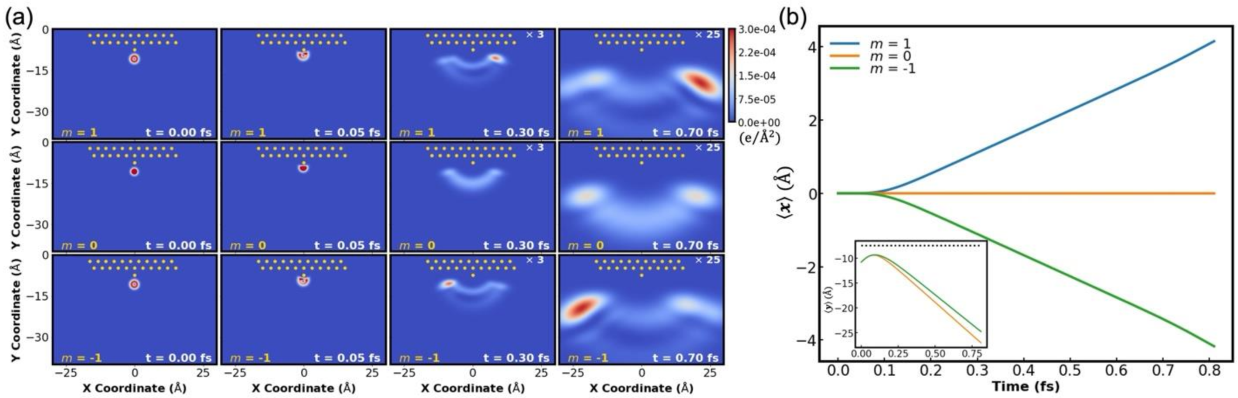

Figure S9. (a) Comparison of scattering trajectories of 2D Gaussian electronic wavepackets, carrying initial angular momenta of $m = +1$ (top row), 0 (middle row), and -1 (bottom row) and colliding head-on with a surface impurity. Snapshots are taken at simulation times of $t = 0$ (left column), 0.05 (second column), 0.3 (third column), and 0.7 (right column) fs. (b) Temporal evolution of the lateral ($\langle x \rangle$, main panel) and vertical ($\langle y \rangle$, inset) components of the position expectation value of the electronic wavepackets depicted in panel (a) with $m = +1$ (blue lines), 0 (orange lines), and -1 (green lines). All other simulation parameters are the same as those used for the $\sigma = 1$ Å wavepacket in main text Fig. 7.

Section 6. Dependence of wavepacket deflection on the repulsive potential parameters

The scattering sites implemented in the simulations presented in the main text induce a repulsive exponential potential of the form $V_{\mathrm{P}}(\boldsymbol{r}) = Ae^{-|\boldsymbol{r}-\boldsymbol{r}_{\mathrm{P}}|/b}$ on the electronic wavepacket, where A is the repulsion strength, b is the interaction length, and $\boldsymbol{r}_{\mathrm{P}}$ is the (instantaneous when dynamic) position of the scatterer. Here, we demonstrate that the qualitative nature of our results is insensitive to the specific choice of potential parameters, within a wide parameter range.

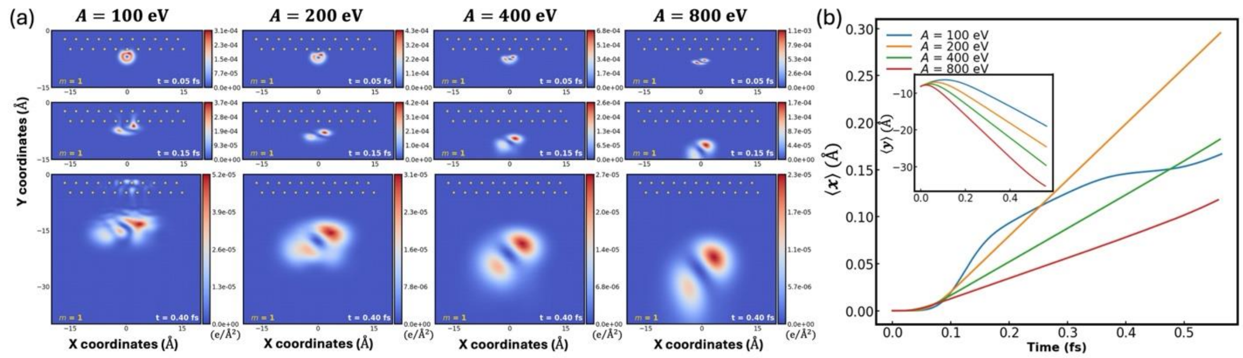

Figure S10. (a) Comparison of head-on collision scattering trajectories of 2D Gaussian wavepackets carrying an initial angular momentum of $m = +1$ with a fixed two-row scatterer surface (augmented by a rear vertical LJ potential) with a scatterer repulsion length of $b = 1$ Å and exponential repulsive strength of $A = 100$ (left column), 200 (second column from the left), 400 (third column from the left) and 800 (right column) eV. Snapshots are taken at a simulation time of 0.05 (top panels), 0.15 (middle panels) and 0.4 fs (bottom panels). (b) Temporal evolution of the lateral ($\langle x \rangle$, main panel) and vertical ($\langle y \rangle$, inset) components of the position expectation value of the electronic wavepackets depicted in panel (a) with $A = 100$ (blue line), 200 (orange line), 400 (green line), and 800 (red line) eV. All other simulation parameters are the same as those used for the $\sigma = 1$ Å wavepacket in main text Fig. 6.

Fig. S10 compares the trajectories of 2D Gaussian electronic wavepackets of initial width of $\sigma = 1$ Å, carrying angular momentum of $m = +1$, and given an initial vertical velocity of $v_y = 30$ Å/fs towards a two-row wall (augmented by a rear vertical LJ potential) with inter-scatterer and inter-row spacings of $d = 3$ Å, lateral inter-row shift of $0.5d$, and a scatterer interaction length of $b = 1$ Å and repulsion strengths of $A = 100$ (blue lines), 200 (orange lines), 400 (green lines) and 800 (red lines) eV. Panel (a) of Fig. S10 shows that all trajectories demonstrate similar

qualitative behavior, where as the interaction strength increases, the wavepacket deflects faster away from the wall (as manifested by its increased distance from the scattering surface at the same simulation time). This is also clearly reflected in the inset of Fig. S10b, showing that higher scatterer repulsion strength results in increased slope (in absolute value) of the expectation value of the wavepacket vertical position as a function of time. Notably, the lateral wavefunction deflection does not show monotonic behavior with repulsion strength, where the lowest value chosen ($A = 100$ eV) allows the wavepacket to penetrate the surface, thus distorting the overall sideways deflection, whereas for the higher values considered ($A = 400$ and 800 eV) the interaction time of the wavepacket with the wall is reduced hence the lateral deflection diminishes with respect to the $A = 200$ eV case used in the simulations presented in the main text.

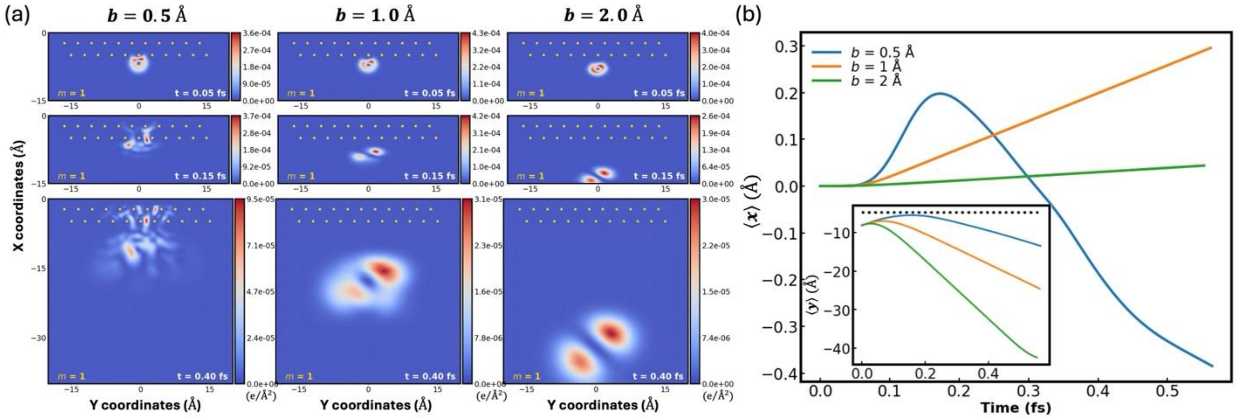

Figure S11. (a) Comparison of head-on collision scattering trajectories of 2D Gaussian wavepackets carrying an initial angular momentum of $m = +1$ with a fixed two-row scatterer surface (augmented by a rear vertical LJ potential) with a scatterer exponential repulsive strength of $A = 200$ eV and repulsion lengths of $b = 0.5$ (left column), 1 (middle column), and 2 Å (right column). Snapshots are taken at a simulation time of 0.05 (top panels), 0.15 (middle panels) and 0.4 (bottom panels) fs. (b) Temporal evolution of the lateral ($\langle x \rangle$, main panel) and vertical ($\langle y \rangle$, inset) components of the position expectation value of the electronic wavepackets depicted in panel (a) with $b = 0.5$ (blue line), 1 (orange line), and 2 (green line) Å. All other simulation parameters are the same as those used for the $\sigma = 1$ Å wavepacket in main text Fig. 6.

Figure S11 compares the 2D Gaussian electronic wavepackets trajectories for a scatterer interaction strength of $A = 200$ eV and interaction lengths of $b = 0.5$ (blue line), 1 (orange line), and 2 (green line) Å (all other simulation parameters are the same as in Fig. S10). For the smallest value considered, the wavepacket penetrates the surface (see top left panel in Fig. S11a and the blue line in the inset of Fig. S11b), undergoing multiple scattering from the two scatterer rows. This results in a complex trajectory that starts, as expected, with an initial sideways deflection to the right, which eventually switches directions to the left (see blue line in Fig. S11b). When increasing the interaction length to $b = 1$ Å, surface penetration reduces (see top middle panel in Fig. S11a and the orange line in the inset of Fig. S11b) and clear consistent sideways deflection to the right is obtained (see orange line in Fig. S11b). Further increase of the interaction length to $b = 2$ Å, reduces the corrugation of the wall and increases its repulsive range thus resulting in accelerated reflection (see top right panel in Fig. S11a and the green line in the inset of Fig. S11b). As a result, the interaction time of the wavepacket with the wall reduces and the sideways deflection diminishes (see green line in Fig. S11b). Altogether, for physically relevant interaction lengths, the qualitative nature of the scattering trajectories is preserved.

References

(1) Kiran, V.; Mathew, S. P.; Cohen, S. R.; Hernández Delgado, I.; Lacour, J.; Naaman, R. Helicenes—A new class of organic spin filter. *Adv. Mater.* **2016**, *28* (10), 1957-1962.

(2) Kettner, M.; Maslyuk, V. V.; Nurenberg, D.; Seibel, J.; Gutierrez, R.; Cuniberti, G.; Ernst, K. H.; Zacharias, H. Chirality-dependent electron spin filtering by molecular monolayers of helicenes. *J. Phys. Chem. Lett.* **2018**, *9* (8), 2025-2030.

(3) Singh, A.-K.; Martin, K.; Mastropasqua Talamo, M.; Houssin, A.; Vanthuyne, N.; Avarvari, N.; Tal, O. Single-molecule junctions map the interplay between electrons and chirality. *Nat. Commun.* **2025**, *16* (1), 1759.

(4) Kettner, M.; Göhler, B.; Zacharias, H.; Mishra, D.; Kiran, V.; Naaman, R.; Fontanesi, C.; Waldeck, D. H.; Sęk, S.; Pawłowski, J.; et al. Spin Filtering in Electron Transport Through Chiral Oligopeptides. *J. Phys. Chem. C* **2015**, *119* (26), 14542-14547.

(5) Kiran, V.; Cohen, S. R.; Naaman, R. Structure dependent spin selectivity in electron transport through oligopeptides. *J. Chem. Phys.* **2017**, *146* (9), 092302.

(6) Kumar, A.; Capua, E.; Kesharwani, M. K.; Martin, J. M.; Sitbon, E.; Waldeck, D. H.; Naaman, R. Chirality-induced spin polarization places symmetry constraints on biomolecular interactions. *Proc. Natl. Acad. Sci. U.S.A.* **2017**, *114* (10), 2474-2478.

(7) Mishra, S.; Mondal, A. K.; Pal, S.; Das, T. K.; Smolinsky, E. Z. B.; Siligardi, G.; Naaman, R. Length-Dependent Electron Spin Polarization in Oligopeptides and DNA. *J. Phys. Chem. C* **2020**, *124* (19), 10776-10782.

(8) Das, T. K.; Tassinari, F.; Naaman, R.; Fransson, J. Temperature-dependent chiral-induced spin selectivity effect: experiments and theory. *J. Phys. Chem. C* **2022**, *126* (6), 3257-3264.

(9) Nguyen, T. N. H.; Rasabathina, L.; Hellwig, O.; Sharma, A.; Salvan, G.; Yochelis, S.; Paltiel, Y.; Baczewski, L. T.; Tegenkamp, C. Cooperative effect of electron spin polarization in chiral molecules studied with non-spin-polarized scanning tunneling microscopy. *ACS Appl. Mater. Interfaces* **2022**, *14* (33), 38013-38020.

(10) Adhikari, Y.; Liu, T.; Wang, H.; Hua, Z.; Liu, H.; Lochner, E.; Schlottmann, P.; Yan, B.; Zhao, J.; Xiong, P. Interplay of structural chirality, electron spin and topological orbital in chiral molecular spin valves. *Nat. Commun.* **2023**, *14* (1), 5163.

(11) Moharana, A.; Kapon, Y.; Kammerbauer, F.; Anthofer, D.; Yochelis, S.; Shema, H.; Gross, E.; Kläui, M.; Paltiel, Y.; Wittmann, A. Chiral-induced unidirectional spin-to-charge conversion. *Sci. Adv.* **2025**, *11* (1), eado4285.

(12) Göhler, B.; Hamelbeck, V.; Markus, T.; Kettner, M.; Hanne, G.; Vager, Z.; Naaman, R.; Zacharias, H. Spin selectivity in electron transmission through self-assembled monolayers of double-stranded DNA. *Science* **2011**, *331* (6019), 894-897.

(13) Xie, Z.; Markus, T. Z.; Cohen, S. R.; Vager, Z.; Gutierrez, R.; Naaman, R. Spin specific electron conduction through DNA oligomers. *Nano Lett.* **2011**, *11* (11), 4652-4655.

(14) Watson, J. D.; Crick, F. H. Molecular structure of nucleic acids: a structure for deoxyribose nucleic acid. *Nature* **1953**, *171* (4356), 737-738.

(15) Drew, H. R.; Wing, R. M.; Takano, T.; Broka, C.; Tanaka, S.; Itakura, K.; Dickerson, R. E. Structure of a B-DNA dodecamer: conformation and dynamics. *Proc. Natl. Acad. Sci. U.S.A.* **1981**, *78* (4), 2179-2183.

(16) Pawlowski, J.; Juhaniewicz, J.; Tymecka, D.; Sek, S. Electron transfer across α-helical peptide monolayers: Importance of interchain coupling. *Langmuir* **2012**, *28* (50), 17287-17294.

(17) Nguyen, T. N. H.; Xue, S.; Tegenkamp, C. Heterochiral Dimer Formation of α-l-and α-d-Polyalanine Molecules on Surfaces. *J. Phys. Chem. C* **2020**, *124* (20), 11075-11080.

(18) Shen, Y.; Chen, C.-F. Helicenes: synthesis and applications. *Chem. Rev.* **2012**, *112* (3), 1463-1535.

(19) Li, T. E.; Nitzan, A.; Sukharev, M.; Martinez, T.; Chen, H.-T.; Subotnik, J. E. Mixed quantum-classical electrodynamics: Understanding spontaneous decay and zero-point energy. *Phys. Rev. A* **2018**, *97* (3), 032105.
